# Use of Telehealth Across Pediatric Subspecialties Before and During the COVID-19 Pandemic

**DOI:** 10.1001/jamanetworkopen.2022.4759

**Published:** 2022-03-31

**Authors:** Lori Uscher-Pines, Colleen McCullough, Michael S. Dworsky, Jessica Sousa, Zach Predmore, Kristin Ray, Anthony Magit, Chris Rivanis, Carlos Lerner, Joy Iwakoshi, Steven Barkley, James P. Marcin, Troy McGuire, Michael-Anne Browne, Craig Swanson, John Patrick Cleary, Erin Kelly, Katie Layton, Lucy Schulson

**Affiliations:** 1 RAND Corporation, Arlington, Virginia; 2 Department of Pediatrics, University of Pittsburgh, Pittsburgh, Pennsylvania; 3 Rady Children’s Hospital San Diego, San Diego, California; 4 Children’s Hospital Orange County, Orange, California; 5 UCLA Mattel Children’s Hospital, Los Angeles, California; 6 Loma Linda University Health, Loma Linda, California; 7 Cottage Children's Medical Center, Santa Barbara, California; 8 UC Davis Children’s Hospital, Sacramento, California; 9 Children’s Hospital Los Angeles, Los Angeles, California; 10 Stanford Children’s Health, Stanford, California; 11 Sutter Children’s Medical Center, Sacramento, California; 12 Children’s Specialty Care Coalition, Sacramento, California; 13 Department of Medicine, Boston University School of Medicine, Boston, Massachusetts

## Abstract

**Importance:**

The identification of variation in health care is important for quality improvement. Little is known about how different pediatric subspecialties are using telehealth and what is driving variation.

**Objective:**

To characterize trends in telehealth use before and during the COVID-19 pandemic across pediatric subspecialties and the association of delivery change with no-show rates and access disparities.

**Design, Setting, and Participants:**

In this cohort study, 8 large pediatric medical groups in California collaborated to share aggregate data on telehealth use for 11 pediatric subspecialties from January 1, 2019, to December 31, 2021.

**Main Outcomes and Measures:**

Monthly in-person and telehealth visits for 11 subspecialties, characteristics of patients participating in in-person and telehealth visits, and no-show rates. Monthly use rates per 1000 unique patients were calculated. To assess changes in no-show rates, a series of linear regression models that included fixed effects for medical groups and calendar month were used. The demographic characteristics of patients served in person during the prepandemic period were compared with those of patients who received in-person and telehealth care during the pandemic period.

**Results:**

In 2019, participating medical groups conducted 1.8 million visits with 549 306 unique patients younger than 18 years (228 120 [41.5%] White and 277 167 [50.5%] not Hispanic). A total of 72 928 patients (13.3%) preferred a language other than English, and 250 329 (45.6%) had Medicaid. In specialties with lower telehealth use (cardiology, orthopedics, urology, nephrology, and dermatology), telehealth visits ranged from 6% to 29% of total visits from May 1, 2020, to April 30, 2021. In specialties with higher telehealth use (genetics, behavioral health, pulmonology, endocrinology, gastroenterology, and neurology), telehealth constituted 38.8% to 73.0% of total visits. From the prepandemic to the pandemic periods, no-show rates slightly increased for lower-telehealth-use subspecialties (9.2% to 9.4%) and higher-telehealth-use subspecialties (13.0% to 15.3%), but adjusted differences (comparing lower-use and higher-use subspecialties) in changes were not statistically significant (difference, 2.5 percentage points; 95% CI, −1.2 to 6.3 percentage points; *P* = .15). Patients who preferred a language other than English constituted 6140 in-person visits (22.2%) vs 2707 telehealth visits (11.4%) in neurology (*P* < .001).

**Conclusions and Relevance:**

There was high variability in adoption of telehealth across subspecialties and in patterns of use over time. The documentation of variation in telehealth adoption can inform evolving telehealth policy for pediatric patients, including the appropriateness of telehealth for different patient needs and areas where additional tools are needed to promote appropriate use.

## Key Points

**Question:**

How has telehealth use during the COVID-19 pandemic varied across pediatric subspecialties, and was telehealth associated with changes in no-show rates and access disparities?

**Findings:**

This cohort study of 8 large pediatric medical groups in California found high variability in telehealth use across subspecialties but no association between telehealth volume and clinic no-show rates. Although overall visits remained stable from the prepandemic to pandemic periods, English-speaking patients and patients of ethnicities other than Hispanic were more likely to be seen via telehealth.

**Meaning:**

Documenting variation in telehealth adoption can inform telehealth policy, including the appropriateness of telehealth for different patient needs and areas where additional tools are needed to promote use.

## Teaser

This cohort study of 8 large pediatric medical groups in California examines trends in pediatric subspecialty telehealth use during the COVID-19 pandemic.

## Introduction

The COVID-19 pandemic led to the rapid expansion of telehealth services. Beginning in March 2020, clinicians quickly transitioned to telehealth to facilitate social distancing and maintain access to care. Before the pandemic, telehealth was not widely adopted in pediatric subspecialty care.^1^ One study^1^ that used commercial claims data showed that telehealth visits with pediatric subspecialists increased from only 1 per 1000 child enrollees in 2019 to 68 per 1000 child enrollees in 2020. This large increase, however, did not entirely offset the steep decrease in in-person visits that occurred in the first year of the pandemic.^2^

This shift in care delivery presented significant challenges for pediatric subspecialists and the families they serve. Although all clinicians faced challenges in transitioning to telehealth, including technology barriers and changes to workflow, pediatric practitioners likely faced additional challenges. For example, children have a more limited ability to provide a history of their symptoms, which may increase clinician reliance on the physical examination for diagnosis and treatment.^3^ Telehealth visits with children also involve additional logistical barriers (eg, inclusion of both pediatric patient and caregiver[s] who may be in different locations).

These challenges with pediatric telehealth may be more pronounced for certain specialties. A 2021 American Academy of Pediatrics review^4^ suggested that telehealth may be well suited for behavioral health concerns, chronic condition management, presurgical visits, and follow-up visits after hospitalizations and emergency department visits. A 2021 Stanford study^5^ suggested that there are fundamental differences in the clinical encounters of different subspecialties (eg, perceived reliance on physical examination) that affect telehealth volume. Although no studies have explored variation in the use of telehealth across different pediatric subspecialties from multiple institutions, studies with adults support this claim. A 2020 study^6^ showed that adoption of telehealth in the early pandemic ranged from 3% to 21% among optometrists, physical therapists, ophthalmologists, and orthopedic surgeons to 55% to 67% among endocrinologists, gastroenterologists, and neurologists.

Although studies^7,8,9,10,11^ on the telehealth experience of 1 pediatric subspecialty at 1 institution are increasingly common, little is known about how different pediatric subspecialties are using telehealth and what is driving variation in adoption and sustained use.^12^ Documentation of this variation is important because unwarranted variation (ie, variation that cannot be explained by differences in patients’ health needs or preferences) can signal the need for quality improvement. A more complete understanding of variation in telehealth use can inform evolving telehealth policy for pediatric patients, including the appropriateness of telehealth for different patient needs and areas where additional tools and strategies are needed to promote appropriate use. To address this gap, we collaborated with 8 large, multispecialty pediatric medical groups in California to characterize trends in telehealth use across pediatric subspecialties and association of delivery change with no-show rates and access disparities.

## Methods

Eight large pediatric medical groups participating in the Children’s Specialty Care Coalition (CSCC) shared data on telehealth use for up to 11 pediatric subspecialties from January 1, 2019, to December 31, 2021. The CSCC is a nonprofit association that represents 2500 pediatric specialists in California. The RAND Corporation Institutional Review Board approved this study and did not require documentation of informed consent because all data were deidentified. This study followed the Strengthening the Reporting of Observational Studies in Epidemiology (STROBE) reporting guideline.

The research team developed a data reporting tool and reporting guidance that participating medical groups used to extract visit data from their electronic health record.^13^ Data elements and definitions were refined after consultation with data officers at participating institutions to facilitate standardized reporting and help ensure data quality.^14^

Following a consensus process, physicians on a CSCC advisory committee selected the 11 specialties for inclusion. Specialties were sampled purposively; we attempted to obtain variation in likely use of telehealth after review of use data among specialists serving adults in 2020.^6^ After data review, the committee selected the specialties of behavioral health, cardiology, dermatology, endocrinology, gastroenterology, genetics, nephrology, neurology, orthopedics, pulmonology, and urology for inclusion.

In June 2021, medical groups submitted aggregated data on completed in-person and telehealth outpatient visits for each subspecialty by month. They also submitted data on total scheduled and completed visits for each subspecialty by month. Visits were defined as those delivered by clinicians (physicians, nurse practitioners, and physician assistants) who were board certified or board eligible in the pediatric subspecialty or by clinicians who predominantly treated children (ie, ≥50% of their patient population was ≤17 years of age). For behavioral health and genetics, we also included visits delivered by psychologists, licensed clinical social workers, and genetic counselors. Medical groups reported total visits and telehealth visits (inclusive of video and audio-only visits) for each month from January 1, 2019, to April 30, 2021. Data included demographic characteristics of the patients (age, race, ethnicity, preferred language, and primary payer) who received care via each modality each month.

**Figure 1.**
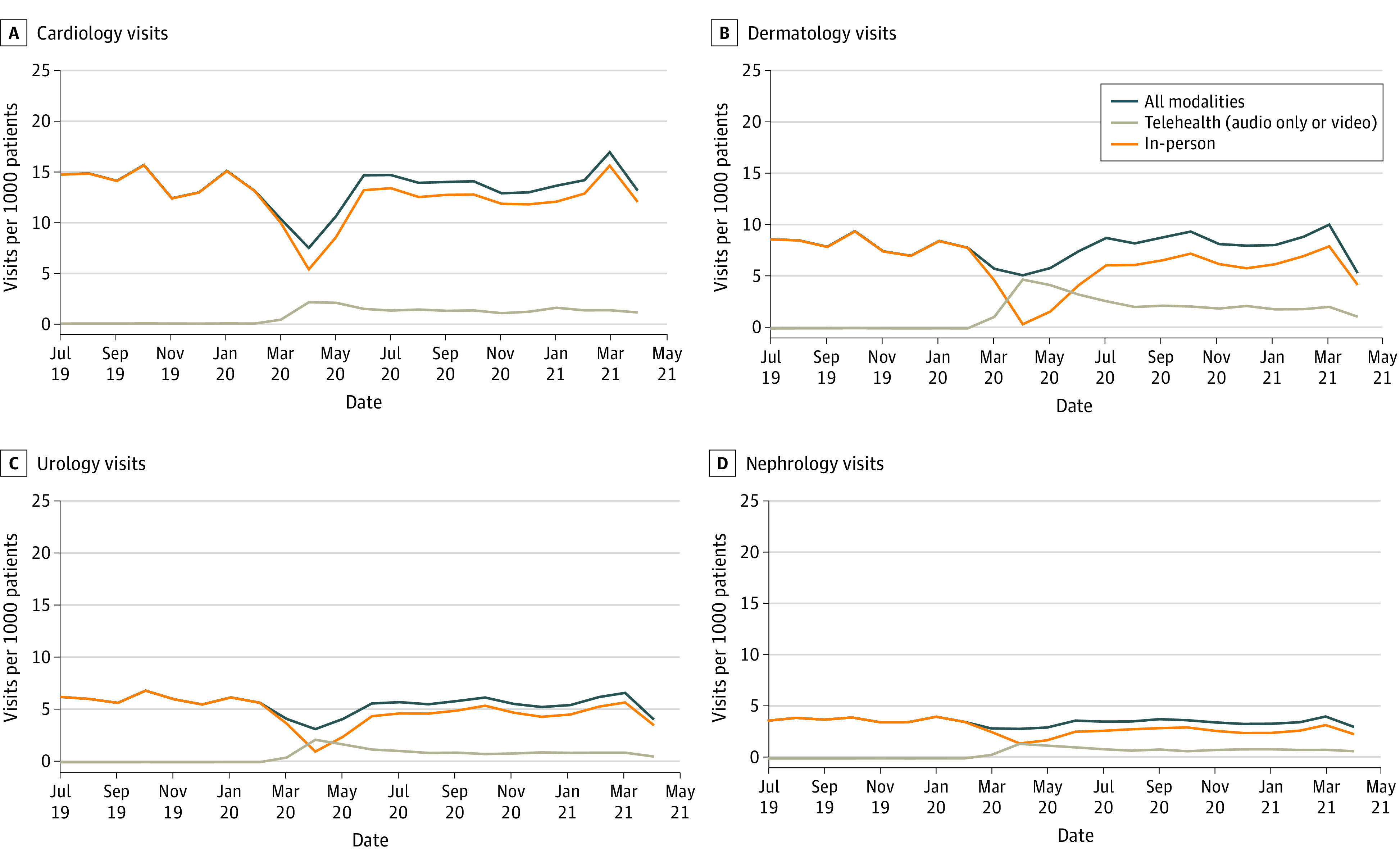
Visits per 1000 Patients by Modality for Select Lower-Telehealth-Use Subspecialties Cardiology and nephrology visits were reported by 8 of 8 organizations (549 306 patients), dermatology visits were reported by 5 of 8 organizations (475 098 patients), and urology visits were reported by 4 of 8 organizations (460 821 patients).

**Figure 2.**
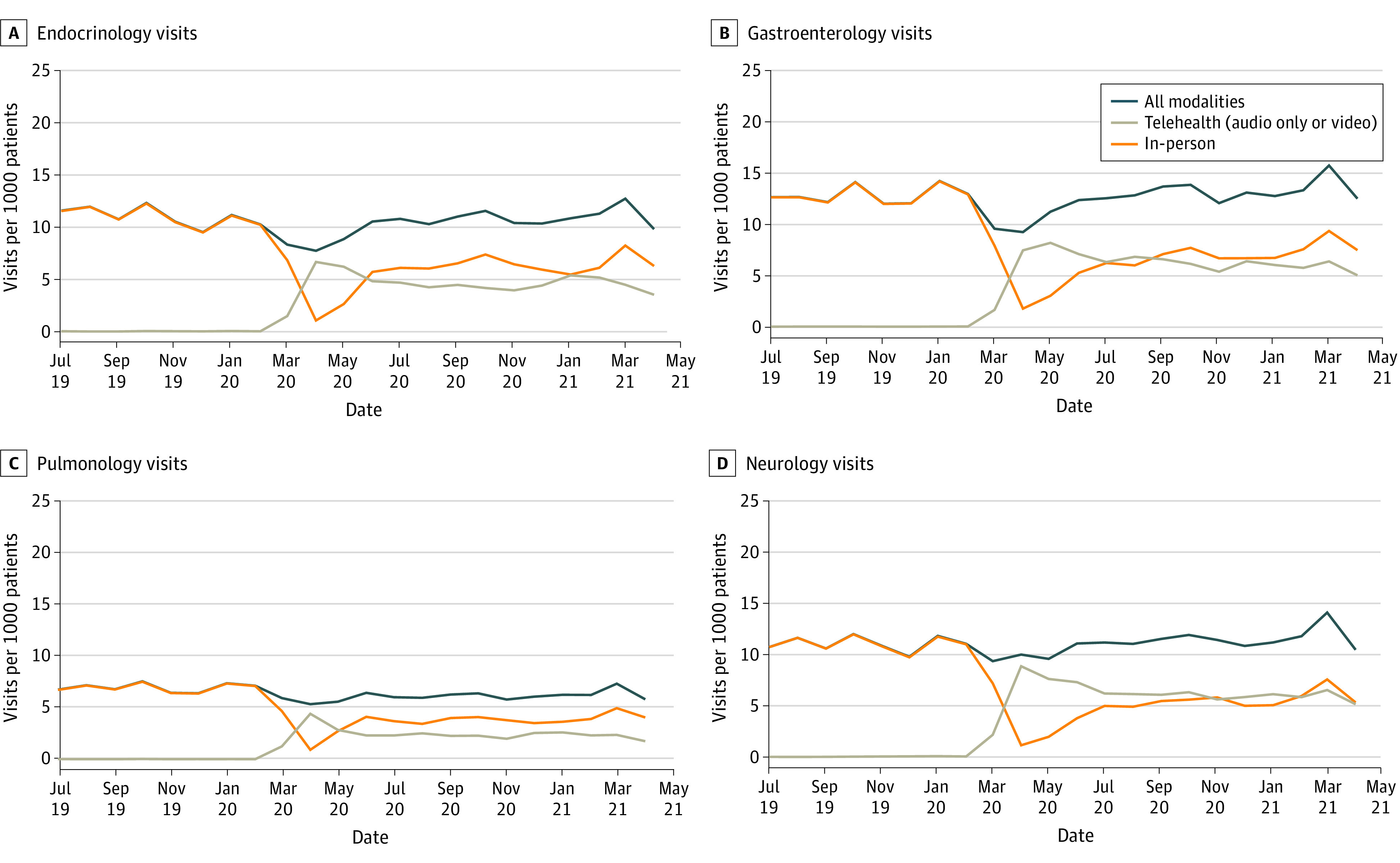
Visits per 1000 Patients by Modality for Select Higher-Telehealth-Use Subspecialties Gastroenterology and endocrinology visits were reported by 8 of 8 organizations (549 306 patients), and neurology and pulmonology visits were reported by 7 of 8 organizations (544 674 patients).

Not all medical groups were able to report visit volumes for all 11 specialties, and 1 medical group was unable to report data on any subspecialties before July 2019 (eTable 1 in the Supplement). Given these limitations in the availability of data, there are some differences across exhibits and across subspecialties within exhibits in the range of months and the number of medical groups included. Summary statistics in Table 1 are calculated using data provided by all medical groups on unique patients treated in 2019. Figure 1 and Figure 2 report trends in visit rates using data only for July 2019 to April 2021 to accommodate the medical group that began reporting in July 2019; trends for each subspecialty reported in Figure 1 reflect visit rates for all medical groups with data for that subspecialty in all months from July 2019 to April 2021. Analyses of no-show rates (Table 2) and patient demographic characteristics by modality (Table 3) use data from March 2019 to April 2021. Data from medical groups with incomplete data were excluded from Table 2 and Table 3.

**Table 1.** Characteristics of Pediatric Patients Across All Specialties at Participating Medical Groups in 2019^a^

Characteristic	No. (%) of patients
Race	
Asian	40 576 (7.4)
Black	20 156 (3.7)
White	228 120 (41.5)
Other^b^	181 053 (33.0)
>1 Race	3439 (0.6)
Unreported	75 962 (13.8)
Ethnicity	
Hispanic	199 987 (36.4)
Ethnicity other than Hispanic	277 167 (50.5)
Unreported	72 152 (13.1)
Age^c^	
≤12 mo	54 324 (9.4)
13 to ≤35 mo	68 597 (11.8)
3 to ≤5 y	72 024 (12.4)
6 to ≤12 y	195 349 (33.7)
13 to ≤17 y	160 050 (27.6)
Preferred language	
English	406 347 (74.0)
Language other than English	72 928 (13.3)
Unreported	70 031 (12.7)
Payer	
None or uninsured	9025 (1.6)
Medicaid	250 329 (45.6)
Other government insurance	30 588 (5.5)
Commercial	258 429 (47.1)
Other	935 (0.2)

^a^ Participating medical groups included Stanford Children’s Health, Rady Children’s Hospital–San Diego Health, Children’s Hospital Los Angeles, UC Davis Children’s Hospital, Children’s Hospital Orange County, Cottage Children’s Medical Center, UCLA Mattel Children’s Hospital, and Loma Linda University Health. All participating medical groups were organizations with a pediatric focus; however, in select cases, organizations had some practitioners who served both children and adults. Visits with individual practitioners who did not predominantly see pediatric patients were excluded.

^b^ Other can include other races not listed (eg, Native American and Pacific Islander) and individuals not identifying as any listed race.

^c^ Column percentages for patient age do not total 100% because approximately 5% of patients were older than 18 years.

**Table 2.** No-Show Rates in the Prepandemic and Pandemic Periods by Subspecialty^a^

Subspecialty	Rate of telehealth use during pandemic period, % (95% CI)	No-show rate, % (95% CI)		Unadjusted change in no-show rate (prepandemic to pandemic period)		Adjusted change in no-show rate (prepandemic to pandemic period)	
		Prepandemic period	Pandemic period	Difference (95% CI)	*P* value	Difference (95% CI)	*P* value
Lower telehealth use							
Orthopedics	5.7 (5.6 to 5.8)	7.9 (7.8 to 8.1)	7.5 (7.3 to 7.7)	NA	NA	−0.5 (−3.6 to 2.5)	.54
Cardiology	10.1 (9.9 to 10.3)	8.6 (8.5 to 8.8)	9.7 (9.5 to 9.9)	NA	NA	0.9 (−1.8 to 3.7)	.43
Urology	17.9 (17.5 to 18.4)	9.8 (9.5 to 10.1)	10.2 (9.9 to 10.5)	NA	NA	0.3 (−3.6 to 4.1)	.78
Nephrology	24.7 (24.2 to 25.3)	9.0 (8.7 to 9.4)	11.7 (11.3 to 12.1)	NA	NA	1.9 (−2.7 to 6.5)	.35
Dermatology	29.0 (28.6 to 29.5)	13.0 (12.7 to 13.3)	11.2 (11.0 to 11.5)	NA	NA	−1.9 (−5.0 to 1.1)	.14
Group mean	13.5 (13.4 to 13.7)	9.2 (9.1 to 9.3)	9.4 (9.3 to 9.5)	0.2 (−1.8 to 2.3)	.78	0.0 (−2.1 to 2.0)	.97
Higher telehealth use							
Pulmonology	38.8 (38.3 to 39.3)	15.1 (14.8 to 15.4)	16.9 (16.6 to 17.3)	NA	NA	1.3 (−3.7 to 6.3)	.53
Endocrinology	42.9 (42.6 to 43.3)	13.1 (12.8 to 13.3)	15.4 (15.2 to 15.7)	NA	NA	2.7 (−1.7 to 7.1)	.19
Gastroenterology	49.9 (49.6 to 50.3)	14.5 (14.3 to 14.7)	15.5 (15.3 to 15.7)	NA	NA	0.7 (−2.6 to 4.1)	.61
Neurology	54.8 (54.4 to 55.1)	11.1 (10.8 to 11.3)	13.3 (13.1 to 13.5)	NA	NA	2.2 (−0.8 to 5.1)	.12
Behavioral health	65.6 (65.0 to 66.1)	11.4 (11.1 to 11.7)	18.2 (17.8 to 18.6)	NA	NA	9.0 (−4.9 to 22.9)	.15
Genetics	73.0 (72.1 to 73.9)	8.7 (8.2 to 9.3)	10.0 (9.4 to 10.6)	NA	NA	1.2 (−2.3 to 4.7)	.41
Group mean	50.2 (50.1 to 50.4)	13.0 (12.9 to 13.1)	15.3 (15.1 to 15.4)	2.3 (−1.0 to 5.6)	.14	2.5 (−0.7 to 5.7)	.10
Difference (higher telehealth − lower telehealth)	36.7 (24.4 to 49.0)	3.8 (0.9 to 6.8)	5.8 (3.0 to 8.7)	2.5 (−1.2 to 6.3)	.15	2.5 (−1.2 to 6.3)	.15

Abbreviation: NA, not applicable.

^a^ Prepandemic period is defined as 12 months from March 1, 2019, to February 28, 2020. Pandemic period is defined as 12 months from May 1, 2020, to April 30, 2021. No-show rates were calculated by taking the ratio of no-show visits (aggregated across medical groups contributing data for each subspecialty) to total scheduled visits (aggregated across medical groups contributing data for each subspecialty).

**Table 3.** Patient Characteristics of Gastroenterology and Neurology Visits During the Prepandemic and Pandemic Periods^a^

Characteristic	Prepandemic visits, No. (%)^b^	Visits during pandemic period, No. (%)			*P* value	
		All	In-person	Telehealth	Prepandemic total vs pandemic total	Pandemic period in-person vs telehealth
**Gastroenterology**						
Overall, No.	80 694	80 953	40 526 (50.1)	40 427 (49.9)	NA	NA
Race						
Asian	6258 (7.8)	6287 (7.8)	2864 (7.1)	3423 (8.5)	<.001	<.001
Black	1861 (2.3)	1989 (2.5)	1146 (2.8)	843 (2.1)		
White	32 546 (40.3)	31 058 (38.4)	15 243 (37.6)	15 815 (39.1)		
Other or not reported^c^	40 058 (49.6)	41 619 (51.4)	21 273 (52.5)	20 346 (50.3)		
Ethnicity						
Hispanic	30 542 (37.8)	29 532 (36.5)	16 083 (39.7)	13 449 (33.3)	<.001	<.001
Ethnicity other than Hispanic	36 813 (45.6)	36 027 (44.5)	15 791 (39.0)	20 236 (50.1)		
Not reported	13 339 (16.5)	15 394 (19.0)	8652 (21.3)	6742 (16.7)		
Preferred language						
English	43 956 (79.5)	45 117 (80.6)	23 955 (76.7)	21 162 (85.7)	<.001	<.001
Non-English language	11 273 (20.4)	10 773 (19.3)	7255 (23.2)	3518 (14.2)		
Other or not reported	59 (0.1)	63 (0.1)	39 (0.1)	24 (0.1)		
Age						
≤35 mo	12 269 (20.5)	12 974 (21.2)	7025 (22.4)	5949 (20.0)	<.001	<.001
3 to ≤5 y	6670 (11.1)	6462 (10.6)	3151 (10.0)	3311 (11.1)		
6 to ≤12 y	18 920 (31.6)	17 882 (29.2)	9052 (28.8)	8830 (29.7)		
13 to ≤17 y	18 020 (30.1)	19 036 (31.1)	9905 (31.5)	9131 (30.7)		
≥18 y	3952 (6.6)	4809 (7.9)	2273 (7.2)	2536 (8.5)		
Other or not reported	0	0	0	0		
Payer						
None or uninsured	3659 (4.5)	559 (0.7)	237 (0.6)	322 (0.8)	<.001	<.001
Medicaid	36 434 (45.2)	39 717 (49.1)	21 585 (53.3)	18 132 (44.9)		
Other government	2439 (3.0)	2111 (2.6)	1105 (2.7)	1006 (2.5)		
Private or commercial	38 091 (47.2)	38 429 (47.5)	17 567 (43.3)	20 862 (51.6)		
Other or not reported	71 (0.1)	137 (0.2)	32 (0.1)	105 (0.3)		
**Neurolog**y						
Overall	70 270	71 036	32 124 (45.2)	38 912 (54.8)	NA	NA
Race						
Asian	4548 (6.5)	4686 (6.6)	2068 (6.4)	2618 (6.7)	<.001	<.001
Black	2040 (2.9)	2014 (2.8)	1053 (3.3)	961 (2.5)		
White	26 105 (37.1)	25 549 (36.0)	11 325 (35.3)	14 224 (36.6)		
Other or not reported^c^	37 591 (53.5)	38 787 (54.6)	17 678 (55.0)	21 109 (54.2)		
Ethnicity						
Hispanic	28 678 (40.8)	28 148 (39.6)	13 228 (41.2)	14 920 (38.3)	<.001	<.001
Ethnicity other than Hispanic	32 091 (45.7)	31 987 (45.0)	12 401 (38.6)	19 586 (50.3)		
Not reported	9501 (13.5)	10 901 (15.3)	6495 (20.2)	4406 (11.3)		
Preferred language						
English	41 671 (80.7)	42 503 (82.7)	21 447 (77.7)	21 056 (88.4)	<.001	<.001
Non-English language	9897 (19.2)	8847 (17.2)	6140 (22.2)	2707 (11.4)		
Other or not reported	74 (0.1)	61 (0.1)	18 (0.1)	43 (0.2)		
Age						
≤35 mo	9506 (15.6)	10 360 (16.8)	6001 (21.2)	4359 (13.1)	<.001	<.001
3 to ≤5 mo	7032 (11.6)	7030 (11.4)	3021 (10.7)	4009 (12.1)		
6 to ≤12 y	20 888 (34.4)	19 824 (32.2)	8747 (30.9)	11 077 (33.4)		
13 to ≤17 y	17 775 (29.3)	18 091 (29.4)	8329 (29.5)	9762 (29.4)		
≥18 y	5543 (9.1)	6184 (10.1)	2177 (7.7)	4007 (12.1)		
Other or not reported	0	0	0	0		
Payer						
None or uninsured	456 (0.6)	425 (0.6)	125 (0.4)	300 (0.8)	.006	<.001
Medicaid	37 251 (53.0)	37 010 (52.1)	18 119 (56.4)	18 891 (48.5)		
Other government	2093 (3.0)	2193 (3.1)	1068 (3.3)	1125 (2.9)		
Private or commercial	30 296 (43.1)	31 221 (44.0)	12 706 (39.6)	18 515 (47.6)		
Other or not reported	177 (0.3)	187 (0.3)	106 (0.3)	81 (0.2)		

Abbreviation: NA, not applicable.

^a^ Gastroenterology visits by patient demographic characteristics reported by 7 of 8 organizations and neurology visits reported by 6 of 8 organizations.

^b^ All in-person visits.

^c^ Other can include other races not listed (eg, Native American and Pacific Islander) and individuals not identifying as any listed race.

On the basis of the recommendation of the advisory committee, we grouped subspecialties into 2 mutually exclusive categories based on the proportion of total visits that were delivered via telehealth from May 2020 to April 2021: lower-telehealth-use subspecialties (≤32% of total visits) and higher-telehealth-use specialties (≥33% of total visits). To describe trends in in-person and telehealth visits, we calculated monthly use rates per 1000 unique patients. To do this, we summed monthly visit counts for each visit type to the subspecialty level (eg, neurology telehealth visits in May 2020) and divided those counts by the total of all unique pediatric patients associated with each medical group in 2019. The denominator used for each subspecialty included only medical groups that contributed visit data for that subspecialty. For example, 7 organizations contributed data on neurology visits, and these organizations treated 544 674 patients in 2019.

### Statistical Analysis

To assess how no-show rates changed for subspecialties with differing levels of telehealth use, we estimated linear regression models using a panel data set at the medical group–specialty–month level of observation. All models included fixed effects for medical group (to adjust for the use of an unbalanced panel of medical groups) and calendar month (to adjust for seasonality in no-show rates). Adjusted changes in no-show rates for individual subspecialties were estimated using a separate regression model for each specialty. Adjusted changes in no-show rates for groups of subspecialties (lower telehealth use and higher telehealth use) were estimated using a separate model for each group; these models included fixed effects for medical group–subspecialty interactions. Finally, the association between telehealth use and adjusted changes in no-show rates was estimated using a difference-in-differences regression model that pooled all subspecialties: we report the coefficient of an interaction term equal to 1 for high-telehealth-use specialties during the pandemic and 0 otherwise. Inference was conducted using SEs clustered on medical group.

To assess whether patients using telehealth during the pandemic were representative of the broader patient population, we compared the demographic characteristics of patients served in person during 12 prepandemic months (March 2019 to February 2020) with those who received in-person and telehealth care during 12 pandemic-period months (May 2020 to April 2021) using χ^2^ tests. We focused on 4 subspecialties: 2 with the largest fraction of telehealth visits as a proportion of total visits (genetics and behavioral health) and 2 with the largest volume of telehealth visits in aggregate during the pandemic period (gastroenterology and neurology). All differences (prepandemic vs pandemic period visits and pandemic period telehealth vs pandemic period in-person visits) were statistically significant because of the large sample size: we accordingly highlighted absolute differences of 5 percentage points or more as clinically significant, as determined post hoc by our research team.

We excluded March and April 2020 from all analyses with significance testing because these months represented the onset of the COVID-19 pandemic when delivery changes were occurring rapidly. Statistical significance was defined as a 2-sided *P* < .05. The analyses were conducted using Stata software, version 17 (StataCorp).

## Results

Participating pediatric medical groups included the largest children’s hospital in California, several other hospitals exclusively dedicated to pediatric care, and the medical system covering the largest geographic region in California. In 2019, these medical groups conducted 1.8 million visits with 549 306 unique patients younger than 18 years (40 576 [7.4%] Asian, 20 156 [3.7%] Black, 228 120 [41.5%] White, 3439 [0.6%] >1 race, and 181 053 [33.0%] other, including other races not listed [eg, Native American and Pacific Islander] and individuals not identifying as any listed race, and 75 962 [13.8%] unreported race; 199 987 [36.4%] Hispanic, 277 167 [50.5%] ethnicity other than Hispanic, and 72 152 [13.1%] unreported ethnicity). A total of 258 429 patients (47.1%) had commercial insurance, 250 329 (45.6%) had Medicaid insurance, and 72 928 (13.3%) preferred a language other than English (Table 1).

Pediatric subspecialties varied with respect to their use of telehealth as a proportion of total visits; furthermore, there was variation in the extent to which telehealth use was sustained at the same level throughout the entire pandemic period. In lower-telehealth-use specialties (cardiology, orthopedics, urology, nephrology, and dermatology), telehealth visits ranged from 5.7% to 29.0% of total visits from May 2020 to April 2021. In higher-telehealth-use subspecialties (genetics, behavioral health, pulmonology, endocrinology, gastroenterology, and neurology), telehealth comprised 38.8% to 73.0% of total visits (Table 2). Telehealth volume in some subspecialties, especially those with low telehealth use, was stable from May 2020 onward, but telehealth volume in other subspecialties was more variable. Dermatology telehealth visits ranged from 4.2 per 1000 patients in May 2020 to 1.1 per 1000 patients in April 2021. Endocrinology visits ranged from 6.2 per 1000 patients in May 2020 to 4.0 per 1000 patients in November 2020 and 5.4 per 1000 patients in January 2021. Visit trends for 8 subspecialties are shown in Figure 1 and Figure 2, with 3 additional subspecialties included in eFigures 1 to 3 in the Supplement.

Visit no-show rates did not change significantly from the prepandemic to the pandemic periods (Table 2). No significant differences were found in overall no-show rates for any subspecialties, including higher-telehealth-use subspecialties, after adjusting for seasonality and medical group–specialty fixed effects. Higher-telehealth-use subspecialties exhibited higher no-show rates in the prepandemic period (13.0% of all visits; 95% CI, 12.9%-13.1%) than lower-telehealth-use subspecialties (9.2% of all visits; 95% CI, 9.1%-9.3%). From the prepandemic to the pandemic periods, no-show rates slightly increased for both lower-telehealth-use (9.2% to 9.4%) and higher-telehealth-use (13.0% to 15.3%) subspecialties, but these changes were not statistically significant (difference, 2.5 percentage points; 95% CI, −1.2 to 6.3 percentage points; *P* = .15). Adjusted differences were generally close (within 0.2 percentage points) to unadjusted differences, although these were also imprecisely estimated and were not significant (difference, 2.5 percentage points; 95% CI, −1.2 to 6.3 percentage points; *P* = .15).

The total number of visits (inclusive of all modalities) was similar in the prepandemic and pandemic periods for gastroenterology, neurology, and genetics, whereas total visits decreased substantially for behavioral health (Table 3; eTable 2 in the Supplement). Nonetheless, there were notable differences in (1) the characteristics of patients who were seen in the prepandemic period (all in person) compared with those who were seen via any modality in the pandemic period and (2) the characteristics of patients who were seen in person compared with via telehealth during the pandemic period. Data on gastroenterology and neurology are reported in Table 3. Data on behavioral health and genetics are reported in eTable 2 in the Supplement.

We observed several differences in the populations of patients who participated in telehealth vs in-person care visits during the pandemic period (Table 3). Hispanic patients, patients who preferred a language other than English, and patients with Medicaid comprised a smaller proportion of patients seen via telehealth compared with in person during the pandemic period. During the pandemic period, Hispanic patients constituted 16 083 in-person visits (39.7%) vs 13 449 telehealth visits (33.3%) in gastroenterology (*P* < .001). Patients who preferred a language other than English constituted 6140 in-person visits (22.2%) vs 2707 telehealth visits (11.4%) in neurology (*P* < .001). Finally, patients with Medicaid constituted 21 585 in-person visits (53.3%) vs 18 132 telehealth visits (44.9%) in gastroenterology (*P* < .001), and 18 119 in-person visits (56.4%) vs 18 891 telehealth visits (48.5%) in neurology (*P* < .001).

## Discussion

We found high variability in adoption of telehealth across subspecialties and in patterns of use over time. We did not identify an association between telehealth volume and clinic no-show rates. Although total visits remained stable from the prepandemic to pandemic periods, English-speaking, non-Hispanic, and commercially insured populations were more likely to be seen via telehealth. Our finding that specialties that rely on interviews (eg, behavioral health and genetics) had higher telehealth use and surgical specialties had lower telehealth use is consistent with prior research.^1,5^ Much of the care provided by surgical and other procedure-based specialties must occur in person.

We had anticipated that subspecialties that depend heavily on physical examinations would be less likely to transition to telehealth, but results were mixed. Cardiology, for example, had low rates of telehealth use. It follows that the diagnosis of cardiac murmurs, for example, depends heavily on a physical examination that may be difficult to perform without an in-person encounter or specialized peripheral devices. Similarly, measuring vital signs, such as blood pressure, may be difficult in a pediatric population if appropriately sized blood pressure cuffs are not readily available.^6^ We note, however, that blood pressure is also relevant for nephrology, which had higher telehealth use. Neurology and gastroenterology had high telehealth use. There are key examination components in these subspecialties, but there may be maneuvers that caregivers can be coached through more readily.

We can only speculate as to why seemingly similar subspecialties differed in their telehealth use. Higher use may be driven by innovations that certain subspecialties were able to adopt. For subspecialties with lower telehealth use, the persistence of in-person visits may also reflect innovations in in-person care (eg, alternative clinic scheduling to reduce the number of people in clinic at one time) rather than challenges with telehealth.

Our estimates of no-show rates do not support the claim that greater telehealth adoption will reduce no-show rates. Limited evidence has come out in 2021 suggesting that no-show rates are lower for telehealth services and that offering convenient telehealth services can improve no-show rates across modalities.^15,16,17^ However, our point estimates suggest that no-show rates increased by more in subspecialties that adopted telehealth more widely, although this study was not designed to identify the causal effect of telehealth adoption on no-show rates. It is plausible that subspecialties expecting a larger increase in no-show rates quickly embraced telehealth during the pandemic, especially given that the higher-telehealth-use subspecialties had higher no-show rates before the pandemic: practitioners might have transitioned to telehealth in part out of a perceived need to maintain access. Furthermore, it is possible that clinicians and patients scheduled telehealth visits during the pandemic despite a preference for in-person visits or known barriers to telehealth. Consequently, no-show rates in both high- and low-telehealth-use subspecialities may look different in nonpandemic times, when other considerations drive scheduling decisions.

As in other studies^18,19,20^ from the first year of the pandemic, we found disparities in telehealth use across populations. For example, patients who preferred a language other than English were more likely to be served in person. Several explanations for disparities appear plausible. First, telehealth platforms and workflows may not adequately incorporate interpreter services. Second, differences may reflect patient preference or practitioner preference or bias. Practitioners as well as clinic staff may not offer telehealth to certain patients because of assumptions around patient preference or (real or perceived) barriers to accessing telehealth.^21,22^ Third, disparities in use may reflect inequities in access to key resources required for telehealth (eg, lack of broadband).^23^ We also note that the clinical implications of our findings are unclear. Some might argue that the care modality does not matter as long as access to care is maintained and quality of care is equivalent, in which case disparities in telehealth use may not be especially important. Others, however, might argue that besides offering convenience, telehealth supports social distancing and helps to protect vulnerable patients from exposure to COVID-19, in which case disparities are worrisome.

### Limitations

This study has several limitations. First, we used aggregate data and could not perform individual-level analyses. Second, we were unable to differentiate between audio-only and video visits. It is likely that telehealth visits in our sample included a mix of audio-only and video visits. We could not differentiate this in our data, but the medical groups reported that scheduled audio-only visits were relatively rare in their systems. Third, to calculate visit rates, we divided visit counts by the number of pediatric patients served in 2019. This approach was necessary because we lacked data on the patient population in subsequent years, but it assumes that the patient population was stable during the study period. Visit rates per 1000 patients should be interpreted with care because it is possible that the pandemic resulted in changes to the patient population (eg, the number of new patients seeking care). Comparisons between the visit rates for telehealth and in-person care should not be affected by this limitation, however. In addition, we used electronic medical reporting of race and preferred language, which has documented limitations.^24^

## Conclusions

This study found that during the COVID-19 pandemic, rates of telehealth use varied across pediatric specialties and English-speaking and non-Hispanic patients were more likely to be seen via telehealth. Although some pediatric subspecialties may have unique challenges delivering telehealth visits of comparable quality to in-person care, others may benefit from using telehealth to improve access for patients. An important next step is to understand how variation impacts clinical outcomes, particularly as hybrid care models become more common in pediatric subspecialty care. The goal of policy makers and practitioners should not necessarily be to reduce the variation in telehealth adoption across subspecialties; rather, a key goal is to ensure that patients and practitioners have the resources and training to support the delivery of hybrid care that incorporates telehealth and in-person care to different degrees as necessary to support high-quality and accessible health care for children.

## Notes

Supplement. **eTable 1.** Details on Missing Data **eTable 2.** Patient Characteristics of Genetics and Behavioral Health Visits During the Pre-Pandemic and Pandemic Periods **eFigure 1.** Genetics Visits per 1,000 Patients by Modality **eFigure 2.** Behavioral Health Visits per 1,000 Patients by Modality **eFigure 3.** Orthopedics Visits per 1,000 Patients by Modality
