# Error in Byline

**DOI:** 10.1001/jamanetworkopen.2022.13283

**Published:** 2022-04-29

In the Original Investigation titled “Use of Telehealth Across Pediatric Subspecialties Before and During the COVID-19 Pandemic,”^1^ published March 31, 2022, the fifth author’s name in the byline should be Zachary Predmore. This article has been corrected.^1^
