# Supplementary material for: Use of Telehealth Across Pediatric Subspecialties Before and During the COVID-19 Pandemic
Source: JAMA Netw Open. 2022 Mar 31;5(3):e224759. doi: 10.1001/jamanetworkopen.2022.4759 (PMC8972035; doi:10.1001/jamanetworkopen.2022.4759)
Supplement: Supplement. — eTable 1. Details on Missing Data eTable 2. Patient Characteristics of Genetics and Behavioral Health Visits During the Pre-Pandemic and Pandemic Periods eFigure 1. Genetics Visits per 1,000 Patients by Modality eFigure 2. Behavioral Health Visits per 1,000 Patients by Modality eFigure 3. Orthopedics Visits per 1,000 Patients by Modality [file jamanetwopen-e224759-s001.pdf]

# Supplementary Online Content

Uscher-Pines L, McCullough C, Dworsky MS, et al. Use of telehealth across pediatric subspecialties before and during the COVID-19 pandemic. *JAMA Netw Open*. 2022;5(3):e224759. doi:10.1001/jamanetworkopen.2022.4759

**eTable 1.** Details on Missing Data

**eTable 2.** Patient Characteristics of Genetics and Behavioral Health Visits During the Pre-Pandemic and Pandemic Periods

**eFigure 1.** Genetics Visits per 1,000 Patients by Modality

**eFigure 2.** Behavioral Health Visits per 1,000 Patients by Modality

**eFigure 3.** Orthopedics Visits per 1,000 Patients by Modality

**This supplementary material has been provided by the authors to give readers additional information about their work.**

© 2022 Uscher-Pines L et al. *JAMA Network Open.*

**eTable 1.** Details on Missing Data

| | Months of Data Available by Medical Group (Total of 28) | | | | | | | |
|---|---|---|---|---|---|---|---|---|
| **Specialty** | **1** | **2** | **3** | **4** | **5** | **6** | **7*** | **8** |
| Behavioral Health | 28 | 12 | 28 | 0 | 28 | 28 | 22 | 28 |
| Cardiology | 28 | 28 | 28 | 28 | 28 | 28 | 22 | 28 |
| Dermatology | 28 | 0 | 0 | 0 | 28 | 28 | 22 | 28 |
| Endocrinology | 28 | 28 | 28 | 8 | 28 | 28 | 22 | 28 |
| Gastroenterology | 28 | 28 | 28 | 28 | 28 | 28 | 22 | 28 |
| Genetics | 28 | 0 | 0 | 28 | 28 | 28 | 22 | 28 |
| Nephrology | 28 | 28 | 28 | 28 | 28 | 28 | 22 | 28 |
| Neurology | 28 | 28 | 0 | 28 | 28 | 28 | 22 | 28 |
| Orthopedics | 28 | 16 | 0 | 0 | 28 | 28 | 22 | 0 |
| Pulmonology | 28 | 28 | 8 | 28 | 28 | 28 | 22 | 28 |
| Urology | 28 | 16 | 0 | 0 | 28 | 28 | 22 | 0 |

*Data available from July 2019-April 2021 (22 months total); data included in overall visit trend figures that focused on July 2019-April 2021

**eTable 2.** Patient Characteristics of Genetics and Behavioral Health Visits During the Pre-Pandemic and Pandemic Periods

| | Visits, No. (%) | | | | P value | |
|---|---|---|---|---|---|---|
| | | Pandemic period visits | | | | |
| **Characteristic** | **Pre-Pandemic visits (all in-person)** | **All** | **In-person** | **Telehealth** | **Pre-pandemic total vs. pandemic total** | **Pandemic period in-person vs. telehealth** |
| **Genetics** | | | | | | |
| **Overall** | 9,099 | 9,132 | 2,467 (27.0) | 6,665 (73.0) | NA | NA |
| **Race** | | | | | | |
| White | 3,162 (34.8) | 3,126 (34.2) | 867 (35.1) | 2,259 (33.9) | 0.5268 | 0.0024 |
| Black | 221 (2.4) | 198 (2.2) | 75 (3.0) | 123 (1.8) | | |
| Asian | 691 (7.6) | 692 (7.6) | 180 (7.3) | 512 (7.7) | | |
| Other/Not reported | 5,025 (55.2) | 5,116 (56.0) | 1,345 (54.5) | 3,771 (56.6) | | |
| **Ethnicity** | | | | | | |
| Hispanic | 3,420 (37.6) | 3,314 (36.3) | 1,087 (44.1) | 2,227 (33.4) | <.01 | <.01 |
| Non-Hispanic | 4,375 (48.1) | 4,302 (47.1) | 1,045 (42.4) | 3,257 (48.9) | | |
| Other/Not reported | 1,304 (14.3) | 1,516 (16.6) | 335 (13.6) | 1,181 (17.7) | | |
| **Preferred Language** | | | | | | |
| English | 6,376 (79.3) | 6,615 (81.8) | 1,830 (74.9) | 4,785 (84.8) | <.01 | <.01 |
| Language other than English | 1,633 (20.3) | 1,427 (17.6) | 614 (25.1) | 813 (14.4) | | |
| Other/Not reported | 28 (0.3) | 43 (0.5) | 0 (0.0) | 43 (0.8) | | |
| **Age** | | | | | | |
| ≤35 months | 2,806 (30.8) | 2,834 (31.0) | 889 (36.0) | 1,945 (29.1) | 0.0446 | <.01 |
| 3 – ≤5 years | 1,536 (16.9) | 1,506 (16.5) | 409 (16.6) | 1,097 (16.4) | | |
| 6 – ≤12 years | 2,281 (25.1) | 2,198 (24.0) | 621 (25.2) | 1,577 (23.6) | | |
| 13 – ≤17 years | 1,184 (13.0) | 1,254 (13.7) | 385 (15.6) | 869 (13.0) | | |
| 18+ years | 1,292 (14.2) | 1,356 (14.8) | 163 (6.6) | 1,193 (17.8) | | |
| Other/Not reported | 0 (0.0) | 6 (0.1) | 0 (0.0) | 6 (0.1) | | |
| **Payer** | | | | | | |
| None/uninsured | 844 (9.3) | 118 (1.3) | 25 (1.0) | 93 (1.4) | <.01 | <.01 |
| Medi-Cal/CCS | 3,754 (41.3) | 4,222 (46.2) | 1,396 (56.6) | 2,826 (42.4) | | |
| Other government insurance | 392 (4.3) | 474 (5.2) | 148 (6.0) | 326 (4.9) | | |
| Private/commercial | 4,098 (45.0) | 4,290 (47.0) | 896 (36.3) | 3,394 (50.9) | | |
| Other/Not reported | 11 (0.1) | 28 (0.3) | 2 (0.1) | 26 (0.4) | | |
| **Behavioral Health** | | | | | | |
| **Overall** | 36,869 | 27,438 | 9,445 (34.4) | 17,993 (65.6) | NA | NA |
| **Race** | | | | | | |
| White | 15,746 (42.7) | 11,610 (42.3) | 4,185 (44.3) | 7,425 (41.3) | <.01 | <.01 |
| Black | 1,955 (5.3) | 1,223 (4.5) | 418 (4.4) | 805 (4.5) | | |
| Asian | 1,634 (4.4) | 1,796 (6.5) | 411 (4.4) | 1,385 (7.7) | | |
| Other/Not reported | 17,534 (47.6) | 12,809 (46.7) | 4,431 (46.9) | 8,378 (46.6) | | |
| **Ethnicity** | | | | | | |
| Hispanic | 17,404 (47.2) | 10,219 (37.2) | 4,950 (52.4) | 5,269 (29.3) | <.01 | <.01 |
| Non-Hispanic | 16,236 (44.0) | 12,516 (45.6) | 3,653 (38.7) | 8,863 (49.3) | | |
| Other/Not reported | 3,229 (8.8) | 4,703 (17.1) | 842 (8.9) | 3,861 (21.5) | | |

© 2022 Uscher-Pines L et al. *JAMA Network Open.*

| | | | | | | |
|---|---|---|---|---|---|---|
| **Preferred Language** | | | | | | |
| English | 29,015 (78.7) | 22,886 (83.4) | 7,208 (76.3) | 15,678 (87.1) | <.01 | <.01 |
| Language other than English | 7,821 (21.2) | 4,462 (16.3) | 2,217 (23.5) | 2,245 (12.5) | | |
| Other/Not reported | 32 (0.1) | 89 (0.3) | 20 (0.2) | 69 (0.4) | | |
| **Age** | | | | | | |
| ≤35 months | 2,778 (7.5) | 2,856 (10.4) | 617 (6.5) | 2,239 (12.4) | <.01 | <.01 |
| 3 - ≤5 years | 3,142 (8.5) | 3,094 (11.3) | 687 (7.3) | 2,407 (13.4) | | |
| 6 - ≤12 years | 16,484 (44.7) | 11,262 (41.0) | 4,066 (43.0) | 7,196 (40.0) | | |
| 13 - ≤17 years | 12,837 (34.8) | 8,804 (32.1) | 3,640 (38.5) | 5,164 (28.7) | | |
| 18+ years | 1,628 (4.4) | 1,422 (5.2) | 435 (4.6) | 987 (5.5) | | |
| Other/Not reported | 0 (0.0) | 0 (0.0) | 0 (0.0) | 0 (0.0) | | |
| **Payer** | | | | | | |
| None/uninsured | 18,652 (50.6) | 4,357 (15.9) | 3,689 (39.1) | 668 (3.7) | <.01 | <.01 |
| Medi-Cal/CCS | 7,258 (19.7) | 6,191 (22.6) | 2,040 (21.6) | 4,151 (23.1) | | |
| Other government insurance | 1,177 (3.2) | 1,282 (4.7) | 211 (2.2) | 1,071 (6.0) | | |
| Private/commercial | 9,368 (25.4) | 12,439 (45.3) | 1,814 (19.2) | 10,625 (59.1) | | |
| Other/Not reported | 414 (1.1) | 3,169 (11.5) | 1,691 (17.9) | 1,478 (8.2) | | |

© 2022 Uscher-Pines L et al. *JAMA Network Open.*

**eFigure 1.** Genetics Visits per 1,000 Patients by Modality

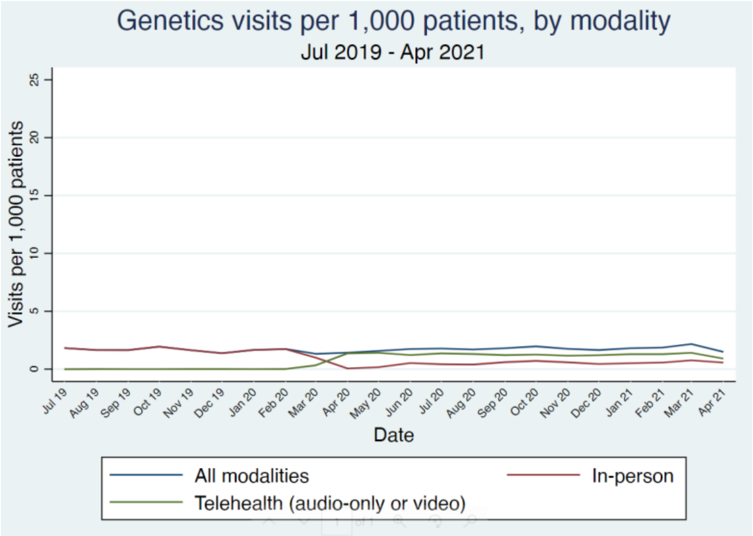


*Genetics visits reported by 6/8 organizations, denominator = 496,609

© 2022 Uscher-Pines L et al. *JAMA Network Open.*

**eFigure 2.** Behavioral Health Visits per 1,000 Patients by Modality

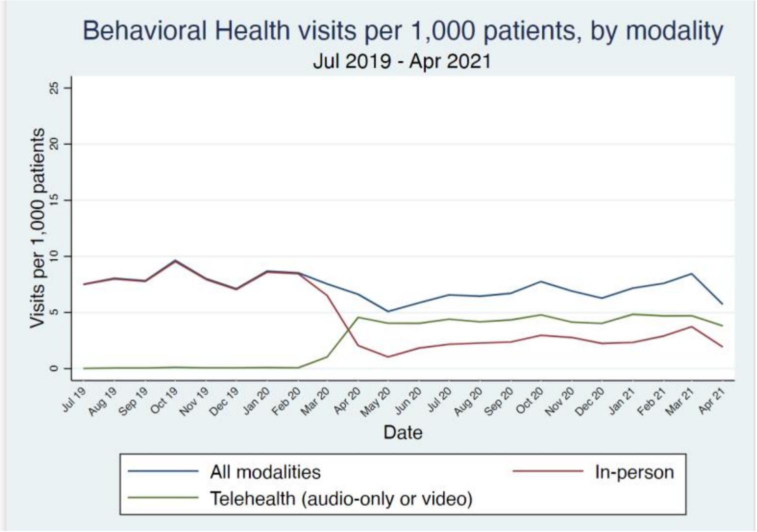


*Behavioral Health visits reported by 5/8 organizations, denominator = 479,730

© 2022 Uscher-Pines L et al. *JAMA Network Open.*

**eFigure 3.** Orthopedics Visits per 1,000 Patients by Modality

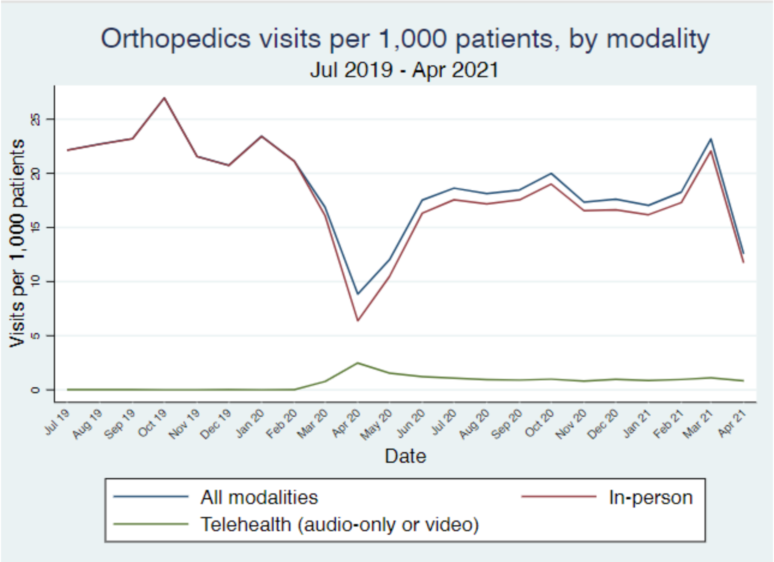


*Orthopedics visits reported by 4/8 organizations, denominator = 460,821

© 2022 Uscher-Pines L et al. *JAMA Network Open.*
